# Azoospermia and reciprocal translocations: should whole-exome sequencing be recommended?

**DOI:** 10.1186/s12610-021-00145-5

**Published:** 2021-11-11

**Authors:** Farah Ghieh, Anne-Laure Barbotin, Julie Prasivoravong, Sophie Ferlicot, Béatrice Mandon-Pepin, Joanne Fortemps, Henri-Jean Garchon, Valérie Serazin, Clara Leroy, François Marcelli, François Vialard

**Affiliations:** 1 Université Paris-Saclay, UVSQ, INRAE, BREED, F-78350 Jouy-en-Josas, France; 2 École Nationale Vétérinaire d’Alfort, BREED, F-94700 Maisons-Alfort, France; 3 Institut de Biologie de la Reproduction-Spermiologie-CECOS, Hôpital Jeanne de Flandre, Centre Hospitalier et Universitaire, F-59000 Lille, France; 4 Service d’Anatomie Pathologique, AP-HP, Université Paris-Saclay, Hôpital de Bicêtre, F-94270 Le Kremlin-Bicêtre, France; 5 Département de Génétique, Laboratoire de Biologie Médicale, CHI de Poissy/Saint-Germain- en-Laye, F-78300 Poissy, France; 6 Service d’Anatomie Pathologique, CHI de Poissy/Saint-Germain-en-Laye, F-78100 Saint- Germain-en-Laye, France; 7 UMR1179, UVSQ, F-78180 Montigny le Bretonneux, France

**Keywords:** Meiotic arrest, Non-obstructive azoospermia, Translocation, Whole-exome sequencing, *TMPRSS9*

## Abstract

**Background:**

Although chromosome rearrangements are responsible for spermatogenesis failure, their impact depends greatly on the chromosomes involved. At present, karyotyping and Y chromosome microdeletion screening are the first-line genetic tests for patients with non-obstructive azoospermia. Although it is generally acknowledged that X or Y chromosome rearrangements lead to meiotic arrest and thus rule out any chance of sperm retrieval after a testicular biopsy, we currently lack markers for the likelihood of testicular sperm extraction (TESE) in patients with other chromosome rearrangements.

**Results:**

We investigated the use of a single nucleotide polymorphism comparative genome hybridization array (SNP-CGH) and whole-exome sequencing (WES) for two patients with non-obstructive azoospermia and testicular meiotic arrest, a reciprocal translocation: t(X;21) and t(20;22), and an unsuccessful TESE. No additional gene defects were identified for the t(X;21) carrier - suggesting that t(X;21) alone damages spermatogenesis. In contrast, the highly consanguineous t(20;22) carrier had two deleterious homozygous variants in the *TMPRSS9* gene; these might have contributed to testicular meiotic arrest. Genetic defect was confirmed with Sanger sequencing and immunohistochemical assessments on testicular tissue sections.

**Conclusions:**

Firstly, *TMPRSS9* gene defects might impact spermatogenesis. Secondly, as a function of the chromosome breakpoints for azoospermic patients with chromosome rearrangements, provision of the best possible genetic counselling means that genetic testing should not be limited to karyotyping. Given the risks associated with TESE, it is essential to perform WES - especially for consanguineous patients.

**Supplementary Information:**

The online version contains supplementary material available at 10.1186/s12610-021-00145-5.

## Résumé

**Contexte:**

Si les réarrangements chromosomiques sont connus pour être à l’origine d’une altération de la spermatogenèse, leur impact dépend fortement des chromosomes impliqués. À l’heure actuelle, la réalisation d’un caryotype et le dépistage des microdélétions du chromosome Y sont les tests génétiques réalisés en première intention chez les patients atteints d’azoospermie non obstructive. S’il est généralement admis que les réarrangements impliquant les chromosomes X ou Y entraînent un arrêt méiotique et réduisent fortement les chances de retrouver des spermatozoïdes après une biopsie testiculaire, nous manquons de marqueurs permettant de définir une probabilité d’extraction de spermatozoïdes testiculaires chez les patients présentant d’autres réarrangements chromosomiques.

**Résultats:**

Nous avons utilisé l’hybridation génomique comparative sur puces (SNP-CGH) et le séquençage entier de l’exome (SEE) pour deux patients présentant une azoospermie non obstructive avec arrêt méiotique, une translocation réciproque: t(X;21) et t(20;22), et sans spermatozoïde retrouvé après biopsie testiculaire Aucune autre anomalie génétique n’a été identifiée chez le patient porteur de la t(X;21) - ce qui suggère que la translocation seule altére la spermatogenèse. En revanche, le patient porteur de la t(20;22), consanguin, présentait deux variants homozygotes délétères dans le gène *TMPRSS9* qui pourraient contribuer à l’arrêt méiotique. Le variant génétique a été confirmé par séquençage Sanger et par immunohistochimie sur des coupes de tissu testiculaire.

**Conclusions:**

Premièrement, nous faisons l’hypothèse d’un impact du défaut du gène *TMPRSS9* sur la spermatogenèse. De plus, en fonction des points de cassures chromosomiques pour les patients azoospermes ayant une translocation réciproque, nous suggérons de ne pas limiter les analyses génétiques à la réalisation d’un caryotype afin d’affiner le conseil génétique. Compte tenu des risques associés à la TESE, il est essentiel de réaliser un SEE en amont et en particulier pour les patients consanguins.

**Mots clés:**

Arrêt méiotique, azoospermie non obstructive, translocation, séquençage de l’exome, TMPRSS9.

## Introduction

The World Health Organization defines azoospermia as the complete absence of spermatozoa in the semen. The prevalence of azoospermia in the male general population is around 1 % [1, 2]. Azoospermia may have an excretory cause (obstruction of the seminal tract, leading to obstructive azoospermia) or a secretory cause (defective sperm production, leading to non-obstructive azoospermia (NOA)) [1, 2]. In both cases, only recourse to an invasive surgical procedure, testicular sperm extraction (TESE) can potentially retrieve spermatozoa and thus enable the couple’s subsequent participation in an *in vitro* fertilization programme [3]. In NOA, the TESE result depends on the three main testicular histological phenotypes: (i) early or late germ cell meiotic arrest (MA), (ii) Sertoli-cell-only syndrome (SCOS), and (iii) hypospermatogenesis with morphological mosaicism. One can also distinguish between pure phenotypes (in which all the seminiferous tubules have the same appearance) and mixed phenotypes (in which the tubules differ in appearance) [4]. TESE is generally negative in cases of pure MA and SCOS, and generally positive in cases of hypospermatogenesis.

Although NOA is likely to have a genetic cause, only a few genetic abnormalities have been specifically identified as recurrent causes of this condition; they include chromosomal aberrations (mainly 47,XXY—Klinefelter syndrome) [5] and Y chromosome microdeletions [6]. Recently, various researchers have used whole-genome analyses (especially array comparative genomic hybridization (CGH) and whole-exome sequencing (WES)) to identify genetic defects associated with spermatogenesis failure or NOA [7–9]. Only defects in Testis-expressed gene 11 (*TEX11)* gene are recurrently identified in NOA patients with MA. The list of gene mutations leading to NOA is growing and thus suggests that a large number of genes are involved in spermatogenesis [10]. Although genetic testing is currently used to help diagnose the aetiology of male infertility, it is not considered to be a prognostic tool for evaluating the likelihood of a positive TESE. In general, we need to find ways of preventing unnecessary TESE in NOA patients with genetic abnormalities. To date, AZFa and/or b microdeletions and a 46,XX karyotype are the only genetic abnormalities that counter-indicate TESE in NOA patients [11]. Conversely, it is now accepted that TESE will be successful in more than 40 % of men with Klinefelter syndrome; this is due to testicular mosaicism and the presence of a few normal 46,XY germ cells in the testis [12].

As with chromosomal rearrangements, the impact of translocations on spermatogenesis probably depends on the chromosomes involved. In cases of azoospermia, only translocations involving the X and Y chromosomes are thought to lead to spermatogenesis failure (due to impairment of the sex vesicle cycle) [13]. However, most andrologists agree that spermatogenesis failure cannot be explained by reciprocal or Robertsonian translocations or inversions alone (especially those involving autosomal chromosomes). The testicular environment might create a highly variable sperm count that ranges from normal to azoospermic. In such a context, a very low sperm count is likely to result from the combination of two genetic defects. With a view to better genetic counselling, the objective of the present study was to evaluate the potential utility of WES prior to TESE for azoospermic patients with reciprocal translocations. To this end, we performed WES for two patients with NOA (due to a reciprocal autosome-autosome translocation in one case and a gonosome-autosome translocation in the other).

## Materials and methods

### Patients

#### Case 1

 A 28-year-old man had failed to conceive after 2 years of unprotected intercourse. Two consecutive sperm analyses revealed azoospermia; this was confirmed after sperm centrifugation. The follicle-stimulating hormone (FSH) and inhibin B levels were 3.7 IU/L and 125 IU/L, respectively. The semen biomarker levels (fructose, citrate, phosphatase acid, and alpha glucosidase) were respectively 102, 126, 3712 and 187 IU/ml. A clinical examination found testicular volumes of 18 and 14 ml. Karyotyping and Y chromosome microdeletion screening revealed a reciprocal translocation: 46,Y,t(X;21)(q10;q10). Despite the karyotyping result and on the basis of the hormone and semen biomarker levels, the patient opted for TESE. No spermatozoa were retrieved, and complete MA at the spermatocyte I stage was diagnosed.

#### Case 2

 A 39-year-old man had failed to conceive after 1.5 years of unprotected intercourse. Two consecutive sperm analyses revealed azoospermia; again, this was confirmed after sperm centrifugation. The FSH and inhibin B levels were 11.5 IU/L and 20 IU/L, respectively. Semen biochemical assays were not performed for this patient. The testicular volume was low (between 6.8 and 7.2 ml). Karyotyping and Y chromosome microdeletion screening revealed a reciprocal translocation: 46,XY,t(20;22)(q11.2;p11.1). Despite the karyotyping result and after an unsuccessful resection for testicular varicocele, the patient opted for TESE. No spermatozoa were retrieved, and a complete MA at the spermatocyte I stage was diagnosed.

### Histological diagnosis

A small piece of the testicular biopsy was fixed in Bouin’s solution (MM France, Brignais, France) for 48 h. Serial sections were prepared from paraffin blocks and stained with haematoxylin and eosin green FCF. All the sections were evaluated by the same expert histologist. Briefly, the mean seminiferous tubule diameter and the tubule wall thickness were measured on 100 seminiferous tubule cross-sections, using an eyepiece micrometre. The sections were classified according to the predominant histological pattern: Sertoli cell-only syndrome (SCOS, i.e. the absence of germ cells within the seminiferous tubules), maturation arrest (i.e. an absence of late-stage spermatogenesis, due to arrest at a particular stage), hypospermatogenesis (i.e. all stages of germ cell are present but in a low numbers), and normal or subnormal spermatogenesis. Furthermore, samples with maturation arrest were classified as “complete MA” when all the seminiferous tubules were affected. We specified the stage of the MA according to the most mature cell type observed during the histological assessment (e.g. MA at the spermatocyte I stage).

### Single nucleotide polymorphism comparative genome hybridization array analysis

In order to detect copy number variations (CNVs) and losses of heterozygosity (LOH, also referred to as runs of homozygosity), we performed array-based single nucleotide polymorphism comparative genome hybridization (SNP-CGH; SurePrint G3 Human Genome CGH + SNP Microarray Kit, 2 × 400 K; Agilent Technologies, Santa Clara, CA 95,051, USA). The array was used according to the manufacturer’s instructions, and commercially sourced male human genomic DNA (Agilent Technologies) was used as the control. Features were extracted from the scanned images using Feature Extraction software (version 4.0, Agilent Technologies) and analysed using the Aberration Detection Method 2 algorithm and CytoGenomics software (https://www.agilent.com/en/genomics-software-downloads; Agilent Technologies). LOH also helped us to analyse the WES results. Furthermore, the total size of all LOH regions (tsLOH) provided us with an opportunity to calculate the inbreeding coefficient for each patient (defined as CsLOH regions/genome size: 3,138 Mb in hg19) according to the American College of Medical Genetics’ standards [14], and to then compare it with the theoretical inbreeding coefficient [15].

### Whole-exome sequencing

Exome sequencing was carried out by Eurofins Genomics (Ebresberg 85,560, Germany) using a HiSeq2500 sequencer (Illumina, San Diego, CA 92,122 USA) and DNA libraries made with Agilent’s SureSelect Exome V6 + UTR Capture Library Kit, according to the manufacturer’s instructions. Sequence reads were trimmed to remove read-through adaptors and low-quality sequences (using fastp [16]) and aligned against the GRCh38 build of the human genome (using the bwa-mem algorithm [17]). After the removal of PCR duplicates using Picard tools (https://broadinstitute.github.io/picard/), the bases were recalibrated using the GATK (v4) tool Baserecalibrator (https://gatk.broadinstitute.org/hc/en-us), as recommended by the Broad Institute Genome Team [18]. Variants were then called with GATK haplotype Caller (https://gatk.broadinstitute.org/hc/en-us), recalibrated with GATK VariantRecalibrator tool (https://gatk.broadinstitute.org/hc/en-us), and annotated with ANNOVAR (https://annovar.openbioinformatics.org/en/latest/) [19].

Only homozygous or compound heterozygous variants with an allele frequency < 1 % in GnomAD and the 1000Genomes project were included. We only retained variants causing insertions/deletions, missense, stop-loss, stop-gain or frameshift mutations, or changes to splice acceptor/donor sites. Synonymous and untranslated 3’ or 5’ regions variants were excluded. We selected missense variants with a high or moderate predicted effect on the encoded protein, as judged with the following prediction algorithms: REVEL (rare exome variant ensemble learner), SIFT (http://sift.jcvi.org/), Polyphen2 (http://genetics.bwh.harvard.edu/pph2/), and M-CAP (http://bejerano.stanford.edu/mcap/). Lastly, in order to identify variants potentially associated with MA, we only considered those in genes strongly or exclusively expressed in the testis or described as being essential for spermatogenesis and meiosis in the literature or in the Gene-Tissue Expression (https://gtexportal.org/home/), Human Protein Atlas (https://www.proteinatlas.org), PubMed or Ensembl databases.

### Sanger sequencing

The variants observed in our analysis were confirmed with Sanger sequencing (carried out by Eurofins Genomics). Primers were designed using Primer 3 Plus software (http://primer3.ut.ee/), and 4 Peaks software (https://nucleobytes.com/4peaks/) was used to read the chromatogram files generated by Eurofins. For each mutation, primers are reported in supplementary material and data (Supplemental Table 1).

### Immunohistochemical assessment

DNMT3B (DNA methyltransferase 3B) and TMPRSS9 (transmembrane protease, serine 9) protein expression levels were assessed on testicular tissue sections from patient 2 and compared with those in a control subject with normal spermatogenesis and who had undergone a testicular biopsy for obstructive azoospermia. We used the Benchmark XT Ventana Roche immunohistochemistry (IHC) system and the XT UltraView Universal DAB Detection kit (Roche Life Science, Penzberg, Germany). To identify and localize the altered proteins in human testicular tissues, 4 μm of paraffin-embedded testis sections from the patient and the control subject were prepared with a microtome. The sections were deposited on IHC slides (SuperFrost Plus type 25 × 75 × 1.0 mm, Thermo Fisher Scientific, Waltham, MA, USA) and then dried at 56^o^C for 24 h to ensure good adhesion to the slide before staining. After the inhibition of endogenous peroxidases, the sections were incubated with the primary antibodies DNMT3B and TMPRSS9 (NBP1-85815 (1/500) and NBP2-30892 (1/100), NovusBio, Centennial, CO, USA) 32 min. After several rounds of washing, the antigen-antibody complex was visualized using the DAB detection system (Roche Life Science, Penzberg, Germany). Slides were counterstained with haematoxylin, dehydrated, and coverslipped for microscopy. Each experiment included negative controls not exposed to the primary antibody. Slides were examined under the microscope with a magnification of x40.

## Results

### Single nucleotide polymorphism comparative genome hybridization array analysis and WES

We did not find any CNVs that could explain the patients’ respective spermatogenesis defects. In particular, we did not find any deletions of the *TEX11* gene –the most frequently altered gene in patients with MA, according to the literature data [20, 21]. The inbreeding coefficient was low (0.70 %) for patient 1 and high (5.38 %) for patient 2. These results prompted us to consider patient 2 as consanguineous and (especially for the latter patient) focus on regions of interest for WES.

For patient 1 (a 46,Y,t(X;21)(q10;q10) carrier), we did not find any SNVs that could have explained the MA. For patient 2 (a 46,XY,t(20;22)(q11.2;p11.1) carrier), and taking account of previously identified regions of interest with loss of heterozygosity, we identified a single homozygous single-nucleotide variant (SNV) in the *DNMT3B* gene (p.E115D, rs761700747) that had not previously been reported in the ExAC database (https://gnomad.broadinstitute.org). Given (i) the absence in the patient 2 of clinical signs of the immunodeficiency-centromeric instability-facial anomalies syndrome 1 (OMIM 242,860) associated with deleterious *DNMT3B* SNVs, and (ii) the high predicted variability associated with this SNV in the various databases (Table 1), we decided to screen the entire exome and to focus on genes that are strongly expressed in the testis. Three homozygous SNVs (p.T4A (rs8100709), p.R74W (rs142862960) and p.T1044I (rs72522121)) were identified in *TMPRSS9* gene. Two of the 3 SNVs were considered to be pathogenic and had a low frequency in general population (at 0.0011 and 0.0032, respectively, according to gnomAD). The data are summarized in Table 1. The variants observed in our analysis were confirmed with Sanger sequencing.

**Table 1:** Homozygous SNPs identified for patient 2, using WES

Gene	Mutation	Type	Frequency (gnomAD)	M-CAP	Revel	SIFT	Polyphen2
TMPRSS9	T4A	homozygous	0.0138	unknown	benign	benign	benign
	R74W	homozygous	0.0011	unknown	benign	deleterious	deleterious
	T1044I	homozygous	0.0032	unknown	deleterious	deleterious	deleterious
DNMT3B	E115D	homozygous	< 0.0001	deleterious	benign	benign	probably deleterious

### Immunohistochemical assessment

**Fig. 1:**
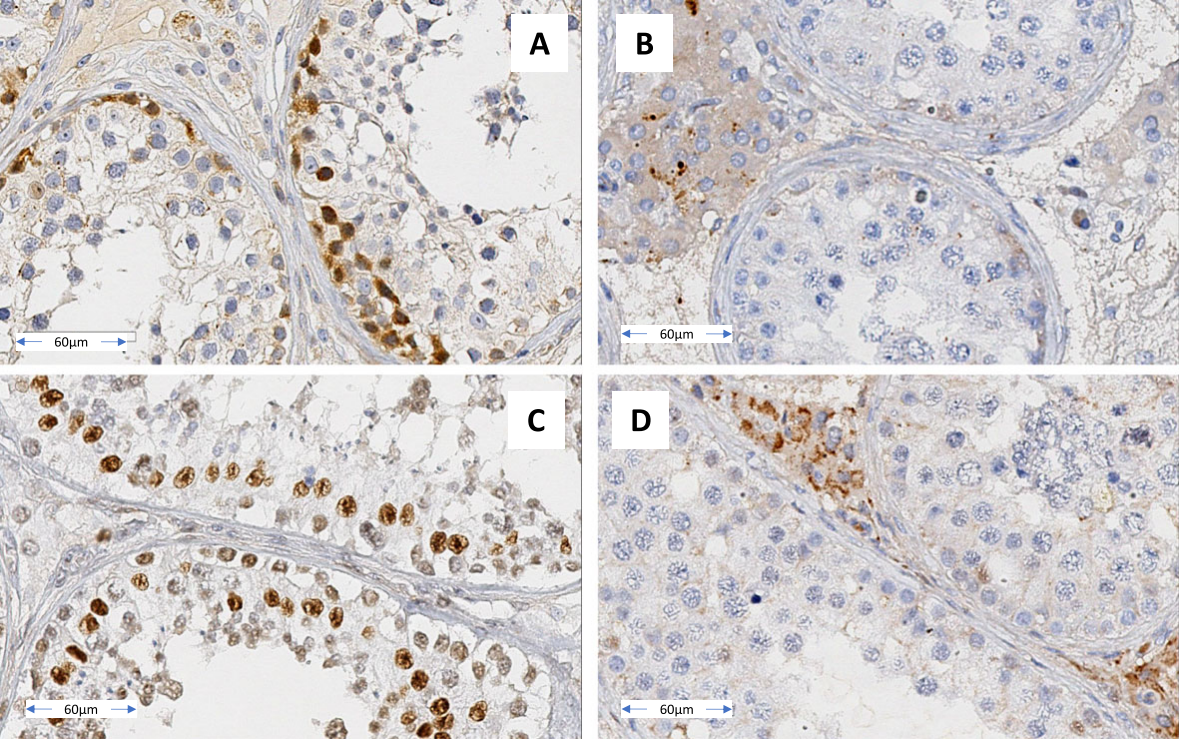
Immunohistochemical assessment of seminiferous tubule expression of TMPRSS9 (**A**: control; **B**: patient 2) and DNMT3B (**C**: control; **D**: patient 2) in the testes. **A** and **C**: normal spermatogenesis, showing normal expression of TMPRSS9 and DNMT3B. **B** and **D**: spermatogenesis arrested at the spermatocyte stage, with no expression of TMPRSS9 or DNMT3B. Staining: the antigen-antibody complex was visualized with the DAB detection system, and slides were counterstained with haematoxylin

In normal spermatogenesis, the TMPRSS9 protein is located in the nuclei of the spermatogonia (control subject: Fig. 1A), and the DNMT3B protein is located in the nuclei of spermatocytes (control subject: Fig. 1C). For patient 2, the seminiferous tubules (containing germs cells arrested at the spermatocyte stage) were not stained (Fig. 1B and D). In contrast, strong DNMT3B staining was detected in the cytoplasm of Leydig cells for the patient 2 but not for the control. These results suggested that the SNVs identified in patient 2 were linked to his testicular phenotype.

## Discussion

### The SNVs’ impact on spermatogenesis

We identified three rare variants for patient 2 and then developed a hypothesis for the possible link between consanguinity, maturation arrest during spermatogenesis, and gene defects. The three *TMPRSS9* mutations all occurred in the isoform ENST00000332578.7, which is strongly expressed in testis. Both TMPRSS9 and DNMT3B proteins are strongly expressed in testes, according to the Gene-Tissue Expression database and the Human Protein Atlas. Although TMPRSS9 mRNA expression has also been reported in the liver and spleen, there are no data on protein levels in these organs. High levels of DNMT3B mRNA expression are also observed in testes. The DNMT3B protein appears to be present in many tissues, with the highest levels in testis and placenta. An IHC assessment of a testicular biopsy from a control subject with a positive TESE and complete spermatogenesis revealed strong TMPRSS9 protein staining in the nuclei of spermatogonia A and B and strong DNMT3B protein staining in spermatocytes. These results were in line with the expression data recorded in the Human Protein Atlas and indicated that TMPRSS9 is expressed earlier than DNMT3B in the seminiferous tubules during normal spermatogenesis. If a defect in the *TMPRSS9* gene does indeed cause MA, it would presumably occur at the spermatogonia stage (i.e. before DNMT3B expression). More generally, the absence of TMPRSS9 is associated with the absence of all the proteins expressed downstream (e.g. DNMT3B); this might explain the absence of protein expression in patient 2’s testicular biopsy. Furthermore, many pathogenic allelic variants in *DNMT3B* have been identified [22, 23], all of which were associated with the immunodeficiency-centromeric instability-facial anomalies syndrome 1 (ICF1) (OMIM 242,860). The variants spanned the entire sequence, and there were no hotspots. Taking into account the great variability in the SNV’s impact predicted by the various databases and in the absence of clinical manifestations of ICF1 syndrome, we conclude that the *DNMT3B* SNV did not have an effect on spermatogenesis. We therefore hypothesize that *TMPRSS9* gene defects (caused here by 2 rare pathogenic SNVs) may impair meiosis and lead to a spermatogenic defect. Further experiments will be necessary to refine our results for TMPRSS9 and to characterize its putative functional role during spermatogenesis; this role has never been studied, even though the gene is known to be mainly or exclusively expressed in the testis. To date, *TMPRSS9* variants have been only reported in a single patient with autism spectrum disorders [24], who was a compound heterozygote for 2 frameshift mutations. Only one homozygous *Tmprss9* knockout mice has been reported in literature, with an exon 2 deletion. [25]. If mice were fertile, a residual *Tmprss9* mRNA expression could be observed; however, no definitive conclusions could be drawn with regard to fertility. In order to understand the impact of TMPRSS9 on spermatogenesis, new null mouse models (i.e. models lacking mRNA expression) should be designed.

### The impact of reciprocal translocation on spermatogenesis

Reciprocal translocations between two autosomes are the most frequent structural chromosomal abnormalities in humans. The prevalence of chromosomal abnormalities is 6.5 times higher in infertile male adults than in newborns [26]. Chromosome screening studies have established the relationship between the sperm count on one hand and the frequencies and types of chromosomal abnormalities on the other. It appears that reciprocal translocations involving only autosomes are more frequent in oligozoospermia, and that those involving gonosomes are associated with severe male infertility and azoospermia [27]. Gonosome-autosome translocations are rare and are divided into three groups, depending on the gonosome involved: Y-autosome translocations (i.e. t(Y;A)), X-autosome translocations (i.e. t(X;A)), and X–Y translocations. Spermatogenetic arrest at the pachytene stage is mainly due to disruption of sex vesicle formation by the quadrivalent observed in all translocation segregations [28]. However, the majority of these patients have severe oligozoospermia and are infertile. For t(Y;A), it has been hypothesized that a smaller translocation segment is associated with more frequent bivalent formation and a higher sperm count [29]. This hypothesis was based on the sperm segregation pattern. In the few patients who showed spermatogenesis, the great majority of spermatozoa were derived from alternate and adjacent segregation. For t(X,A), the interpretation was more complicated (as in our present case): various breakpoints were described, and the sperm segregation pattern was high variable [30]. The size of the segment involved in the translocation does not appear to be related to the TESE result. Although autosome (chromosome 1) breakpoints and X chromosome breakpoints are unusually frequent in male infertility [31], the breakpoint profile does not have prognostic value for TESE. Nevertheless, it has been suggested that the presence of an acrocentric chromosome is associated with a poor prognosis [32]. It has also been reported that a poor TESE prognosis is associated with the XY body in rearrangements involving an acrocentric chromosome [33] and an abnormally high frequency of breakpoints on an acrocentric p-arm in infertile patients [31].

### The “two genetic defects” hypothesis

Taken as a whole, these data suggest that the t(X;A) alone could explain MA in patient 1 (with idiopathic azoospermia and normal testicular volume). This hypothesis was strengthened by the absence of a pathological variant in the WES analysis. In contrast, the reciprocal translocation alone could not explain the MA for patient 2. However, the combination of the reciprocal translocation with the 3 rare variants of *TMPRSS9* (2 of which were considered to be deleterious in predictive databases) could probably explained the spermatogenesis phenotype. These observations strengthen the “two genetic defects” hypothesis for spermatogenesis failure, as has been seen for several other pathologies [34].

In view of the present two cases, the possible impact of a reciprocal translocation on spermatogenesis should be discussed more widely during genetic counselling. Should TESE be suggested, therefore, in patients with a reciprocal translocation? In such a case, one must take account of additional genetic abnormalities. In order to evaluate the probability of successful TESE, we suggest that WES could be performed after considering the chromosome involved, the chromosome breakpoints, and the patient’s family medical history. As described above, t(X;A) was probably the main cause of MA in patient 1. Considering this reciprocal translocation and the high probability of inheriting unbalanced chromosomes (due to translocation malsegregation), the probability of sperm retrieval with TESE is probably very low; this point should be discussed with the patient prior to surgery. We now require more data on when TESE should be ruled out completely. In contrast, the reciprocal translocation alone could not explain patient 2’s testicular phenotype. If the inbreeding coefficient is high, WES should be considered - even though the scarcity of literature data on the *TMPRSS9* gene prevents us drawing clear conclusions about TESE.

### Limitations of the WES approach

WES provides an opportunity to map defects in genes involved in early or late germ cell meiotic arrest. However, a number of complicating factors must be borne in mind: the need for informed consent from the patient, the requirement for pre- and post-test genetic counselling, the complexity of data interpretation, and the cost. In order to limit the complexity and reduce the costs, we decided to focus solely on genes primarily or exclusively expressed in the testis; this would hopefully avoid the fortuitous discovery of gene defects associated with other pathologies (such as cancer) to limit patient feedback and the implications for the patient’s family. Even though WES data are still time-consuming to interpret, the development of novel software tools should soon facilitate the identification and interpretation of all genetic variants. Although the present study might constitute the first step in a larger study, we would nevertheless consider implementing whole-genome sequencing as a replacement for WES and karyotyping.

## Conclusion

Prior to TESE, genetic counselling is important for patients with a reciprocal translocation: discussion of the high probability of inheriting unbalanced chromosomes, and evaluation of the family history. However, our case reports also suggest that WES should be recommended for consanguineous patients, in order to better define the likelihood of sperm retrieval and with a focus on genes primarily or exclusively expressed in the testis. Furthermore, we suggest that deleterious *TMPRSS9* variants impact spermatogenesis – although further data are required.

## Supplementary Information

**Additional file 1 **: **Supplemental Table 1**. Primers used for Sanger sequencing in this study.

## Data Availability

All supporting data are available.

## Abbreviations

AZF: Azoospermia factor
CGH: Comparative genome hybridization
CNV: Copy number variation
TsLOH: Total size of all LOH regions
DNMT3B: DNA methyltransferase 3B
FSH: Follicle-stimulating hormone
LOH: Loss of heterozygosity
MA: Meiotic arrest
Mb: Megabase
NOA: Non-obstructive azoospermia
SCOS: Sertoli-cell-only syndrome
SNP: Single nucleotide polymorphism
SNV: Single nucleotide variant
TEX11: Testis-expressed gene 11
TESE: Testicular sperm extraction
TMPRSS9: Transmembrane protease, serine 9
WES: Whole-exome sequencing
