# Supplementary material for: Azoospermia and reciprocal translocations: should whole-exome sequencing be recommended?
Source: Basic Clin Androl. 2021 Nov 11;31:27. doi: 10.1186/s12610-021-00145-5 (PMC8582189; doi:10.1186/s12610-021-00145-5)
Supplement: Supplementary file 1 — Additional file 1 : Supplemental Table 1. Primers used for Sanger sequencing in this study. [file 12610_2021_145_MOESM1_ESM.docx]

**Table S1**: Primers used for Sanger sequencing in this study

| **Primer** | **Sequence** | | | |
| --- | --- | --- | --- | --- |
|  | **Forward** | **Reverse** | **PCR product size** | **Annealing temperature** |
| TMPRSS9-1 | **CCCGCCGTTTTTAAGGGTATAG** | **TCACCCCAGCTGTGAAAACT** | 502pb | 58.6 |
| TMPRSS9-2 | **GGGGACTGTCATTTCACCGT** | **CCAAACACAGACCACCCTCA** | 571pb | 59.9 |
| TMPRSS9-3 | **TTTCCACTCCACAGCCGTTT** | **AAATGCATCCCCATGACCCA** | 574pb | 59 |
| DNMT3B | **GTCCCACCTCATCACCTGTT** | **GGACTCGTCCACATGGTTG** | 250pb | 58.5 |
